# Incidence of surgical site infections cannot be derived reliably from point prevalence survey data in Dutch hospitals

**DOI:** 10.1017/S0950268816003162

**Published:** 2017-01-09

**Authors:** A. P. MEIJS, J. A. FERREIRA, S. C. DE GREEFF, M. C. VOS, M. B. G. KOEK

**Affiliations:** 1 Centre for Infectious Disease Control, Department of Epidemiology and Surveillance, National Institute of Public Health and the Environment (RIVM), Bilthoven, The Netherlands; 2 Department of Statistics, Informatics and Modelling, National Institute of Public Health and the Environment (RIVM), Bilthoven, The Netherlands; 3 Department of Medical Microbiology and Infectious Diseases, Erasmus MC, Rotterdam, The Netherlands

**Keywords:** Incidence, prevalence, surgical site infections, surveillance

## Abstract

Thorough studies on whether point prevalence surveys of healthcare-associated infections (HAIs) can be used to reliably estimate incidence of surgical site infections (SSIs) are scarce. We examined this topic using surveillance data of 58 hospitals that participated in two Dutch national surveillances; HAI prevalence and SSI incidence surveillance, respectively. First, we simulated daily prevalences of SSIs from incidence data. Subsequently, Rhame & Sudderth's formula was used to estimate SSI incidence from prevalence. Finally, we developed random-effects models to predict SSI incidence from prevalence and other relevant variables. The prevalences simulated from incidence data indicated that daily prevalence varied greatly. Incidences calculated with Rhame & Sudderth's formula often had values below zero, due to the large number of SSIs occurring post-discharge. Excluding these SSIs, still resulted in poor correlation between calculated and observed incidence. The two models best predicting total incidence and incidence during initial hospital stay both performed poorly (proportion of explained variance of 0·25 and 0·10, respectively). In conclusion, incidence of SSIs cannot be reliably estimated from point prevalence data in Dutch hospitals by any of the applied methods. We therefore conclude that prevalence surveys are not a useful measure to give reliable insight into incidence of SSIs.

## INTRODUCTION

A surgical site infection (SSI) is a severe surgical complication and is among the most frequently reported types of healthcare associated infections (HAIs) [1]. SSIs are associated with increased morbidity and mortality, as well as a prolonged hospital stay and a high number of hospital readmissions [2–4]. Active surveillance has proved to be an effective tool in infection control programmes [5–9], although the risk reduction attributed to surveillance varies [8, 10, 11]. The gold standard for SSI surveillance is prospective incidence surveillance [12, 13], which gives accurate and detailed information on the occurrence of new cases within a standardized follow-up period. However, in order to provide reliable estimates it typically requires prolonged data collection. Recently, an increasing number of countries as well as the American and European Centers for Disease Control and Prevention (CDC and ECDC) have started to include prevalence surveys in their HAI surveillance programmes [14, 15]. Prevalence surveys measure the proportion of infections at one point in time (or over a short period) in all patients hospitalized at that time. Prevalence surveys are attractive because they are less labour-intensive and cheaper compared to incidence surveillance. On the other hand, prevalence surveys are less specific since they usually include all types of HAI. When performed regularly, however, they can be used to visualize trends in the occurrence of infections or to evaluate infection control programmes [16]. Despite the different objectives of the two surveillance methods, several studies have attempted to convert HAI prevalence into incidence. For this purpose, some formulas have been developed [17–19], which are based on the relationship between incidence and prevalence via the estimated duration of the infection. The most frequently used formula is that of Rhame & Sudderth [13, 20–23], which, however, has not been extensively applied nor studied for calculating incidence of SSIs [15, 21, 24].

In the Dutch national surveillance network on nosocomial infections (PREZIES), SSI incidence surveillance as well as biannual point prevalence surveys of all HAIs are carried out. Recently, it was proposed to use the results from the prevalence surveys to estimate SSI incidence, in order to substantially reduce the national burden of HAI surveillance. As available evidence regarding the use of prevalence surveys for this purpose was insufficient in The Netherlands, in the present study we aim to investigate whether SSI incidence can be reliably predicted from SSI prevalence data reported by Dutch hospitals.

## METHODS

### SSI incidence surveillance

The SSI incidence surveillance of PREZIES is an ongoing programme in which hospitals participate voluntarily. Information concerning patient and operation characteristics, risk factors for SSIs and the occurrence of an SSI are collected. The definitions used to diagnose SSIs are based on the definitions of ECDC and CDC [25–27]. As a large proportion of SSIs develop after discharge from the hospital, post-discharge surveillance is necessary to detect these infections. A more detailed description of the incidence surveillance has been published previously [8, 28, 29].

### HAI prevalence surveys

PREZIES HAI prevalence surveys are performed twice a year, in March and October. All inpatients admitted to the hospital before the day of the survey and aged ⩾1 year are included. The collected data include patient characteristics as well as risk factors for HAIs and the presence of any type of HAI. The definitions used to diagnose SSIs related to the current hospital stay are the same as those used in the incidence surveillance. For SSIs present at admission (and therefore related to a previous hospitalization) diagnosis is based on patient history (instead of based on surveillance definitions) and infections are reported also when they are already cured at the day of the survey. The methods used in the HAI prevalence survey have been described in detail elsewhere [30].

### Data selection

We included data from hospitals participating simultaneously in both surveillance programmes in the years 2007–2011, linking cumulative SSI incidence to the prevalence in each hospital per year. Specialities with fewer than 20 surgeries in either surveillance programme were excluded. No outbreaks of SSIs were reported by the hospitals during the study period. Cumulative incidence was calculated by dividing the number of operations resulting in an SSI by the total number of operations performed per surveillance year. Prevalence of SSIs was calculated as the number of SSIs (active or under treatment and related to the current hospital stay) at the time of the survey, divided by the total number of surgical patients in the hospital at the time of the survey. When hospitals participated in two prevalence surveys in the same year, combined prevalence was calculated using the total number of surgical patients hospitalized during both surveys. SSIs present at admission were excluded in the prevalence surveys, as diagnosis is not based on surveillance definitions and because these patients did not necessarily underwent surgery during their readmission and may therefore not be included in the denominator (total number of surgical patients in the hospital).

For our analyses, we produced three datasets aggregated at hospital level. The first, dataset I, included incidence based on all reported SSIs. Dataset II included incidence based on SSIs diagnosed during the initial hospital stay only. Finally, dataset III was based on prevalence data and included only SSIs diagnosed during the initial hospital stay.

### Data analysis

First, we assessed the variability of daily point prevalence rates within 1 month. For this purpose, daily prevalence of SSIs was simulated from incidence data for each day of the month in which the prevalence survey might be performed. Only SSIs detected during the initial hospital stay were included (dataset II). Daily prevalence was calculated both at the hospital and the national level.

Second, we used the formula of Rhame & Sudderth to estimate annual SSI incidence from prevalence (dataset III) [17]. The relationship between incidence (I) and prevalence (P) in this method is
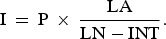


Applied to our study, LA represents the mean length of hospitalization of all patients, LN is the mean length of hospitalization of patients who acquire an SSI, and INT is the mean interval between hospital admission and onset of the SSI.

Since these values could not be derived from the prevalence surveys, they were calculated based on the incidence surveillance. We included, for each hospital, only the specialities that were registered in both surveillance programmes. The values were then calculated taking into account the distribution of patients over the different specialities in the prevalence survey. The accuracy of Rhame & Sudderth's formula was evaluated by comparing the estimated incidence to the observed incidence. The analysis was performed twice, first using incidence based on all SSIs (dataset I) and thereafter including only SSIs detected during initial hospital stay (dataset II).

In the third part of our analyses we developed a linear model to predict SSI incidence from prevalence and other relevant variables including LA, LN, INT, LN minus INT, hospital type, gender, median age and wound class. Because of assumed differences in infection risk between hospitals, hospitals were assigned a random effect. The model was fitted twice, using both incidence of all SSIs (dataset I) and of SSIs detected during initial hospital stay only (dataset II). Because the relationship between incidence and the predictor variables was more likely to be multiplicative than additive, the prediction models adopted were linear on log-transformed variables, but the predictions of the log-incidence were transformed back to yield predictions of incidence ‘on the normal scale’. The predictive performance of the models was assessed by leave-one-out cross-validation [31]. We quantified the performance of the models by the model's proportion of explained variance and by looking at the distribution of the percent difference between the predicted and observed incidence:
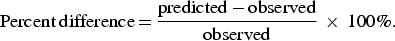


The analyses of the incidence prediction models were performed with R statistical software v. 3.0.1 (R Foundation, Austria). All other analyses were performed with SAS v. 9.3 (SAS Institute Inc., USA).

## RESULTS

**Fig. 1.**
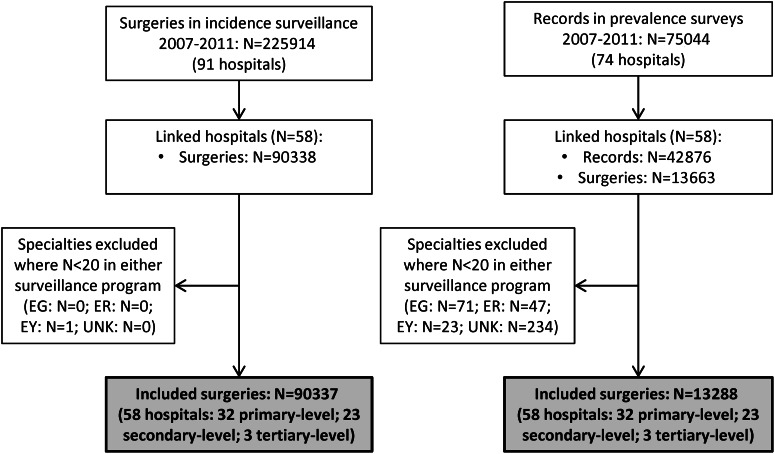
Flowchart of data inclusion. EG, Endocrine glands; ER, ears, nose and throat; EY, ophthalmology; UNK, unknown speciality.

Fifty-eight hospitals that participated in both surveillance programmes for 1–5 years were matched. Figure 1 shows the flowchart of the data inclusion process. Of the 90 337 included surgeries from the incidence surveillance (ranging from 4613 in 2007 to 37 246 in 2011) 2502 SSIs were observed, of which 838 (33%) were diagnosed during initial hospitalization. We included 13 288 surgical patients from the prevalence surveys (ranging from 721 in 2007 to 4258 in 2011) of which 517 had an SSI related to the current hospital stay at time of the survey. Table 1 presents the number of surgical patients, SSIs, lengths of stay and patient characteristics per speciality. The results are presented separately for the dataset including all SSIs (dataset I) and the dataset including SSIs diagnosed during the initial hospital stay only (dataset II). The length of stay of the total patient population (LA) is the same in both datasets, but in dataset II the value of LN is higher and the value of INT fell, as a result of the selection of SSIs during admission only.

**Table 1.** Number of surgical patients, SSIs and patient characteristics in the incidence surveillance and prevalence surveys, per speciality

	Speciality												
	BL	BR	FGO	GI	HCV	MGO	MS	NS	OBS	PV	RS	RU	SS
***Incidence surveillance***													
Surgical patients (*N*)	871	6264	2967	9900	1375	99	57 254	1376	9058	959	54	127	33
**All SSIs**													
SSI, % (*N*)	2·8 (24)	3·7 (231)	1·1 (33)	8·5 (845)	4·1 (56)	1·0 (1)	1·8 (1055)	1·2 (17)	1·4 (125)	11·6 (111)	1·9 (1)	1·6 (2)	3·0 (1)
LA (days)	1·7	2·0	3·8	7·7	10·9	0·5	6·8	4·2	4·7	10·9	10·7	8·0	3·5
LN (days)	3·0	3·2	6·1	21·4	17·9	0·0	12·9	12·5	4·7	16·8	45·0	8·5	0·0
INT (days)	14·0	14·9	12·5	12·3	36·0	15·0	41·6	18·5	14·3	25·2	24·0	12·5	6·0
**SSIs during hospital stay**													
SSI, % (*N*)	0·3 (3)	0·3 (16)	0·2 (6)	5·3 (526)	1·5 (21)	0·0 (0)	0·4 (216)	0·3 (4)	0·1 (7)	4·0 (38)	1·9 (1)	0·0 (0)	0·0 (0)
LA (days)	1·7	2·0	3·8	7·7	10·9	0·5	6·8	4·2	4·7	10·9	10·7	8·0	3·5
LN (days)	9·7	11·1	13·0	30·4	32·2	−	34·6	38·3	15·1	29·6	45·0	−	−
INT (days)	3·0	6·4	5·7	11·0	13·8	−	13·6	19·5	7·6	15·1	24·0	−	−
**Patient characteristics**													
Gender (% men)	11·8	1·6	0·0	41·8	80·5	100·0	32·4	50·9	0·0	66·6	59·3	58·3	3·0
Age, median, years, IQR	58 (49–67)	59 (49–69)	48 (42–56)	60 (44–72)	72 (65–77)	24 (9–60)	71 (63–78)	64 (54–73)	31 (28–35)	72 (64–78)	64 (47–71)	66 (56–74)	51 (38–55)
Wound class (%)													
<3	95·9	98·2	98·4	82·1	95·4	99·0	98·9	99·5	94·7	95·0	94·4	99·2	97·0
⩾3	0·5	0·2	0·1	15·4	0·7	1·0	0·1	0·3	0·1	0·7	5·6	0·0	0·0
UNK	3·7	1·6	1·6	2·5	3·9	0·0	1·0	0·2	5·2	4·3	0·0	0·8	3·0
***Prevalence survey***													
Surgical patients (*N*)	104	253	860	2853	1198	249	4731	494	492	387	462	702	503
SSI, % (*N*)	2·9 (3)	1·6 (4)	0·9 (8)	9·1 (261)	4·5 (54)	0·8 (2)	2·4 (115)	2·2 (11)	0·4 (2)	5·9 (23)	1·1 (5)	2·4 (17)	2·4 (12)
**Patient characteristics**													
Gender (% men)	37·5	2·0	0	50·6	69·2	100	38·5	48·0	0	54·3	59·1	71·1	49·1
Age, median, years, IQR	60 (50–73)	56 (46–71)	48 (30–64)	65 (53–75)	70 (61–77)	69 (62–76)	71 (60–81)	60 (48–72)	32 (28–36)	70 (61–77)	59 (41–69)	69 (60–76)	65 (49–78)
Wound class (%)													
<3	93·3	93·3	85·5	71·1	89·7	83·9	85·3	96·0	93·1	88·9	87·5	90·0	51·3
⩾3	1·0	3·6	6·1	20·7	2·9	1·6	10·0	2·6	2·0	4·9	5·2	2·6	39·4
UNK	5·8	3·2	8·5	8·2	7·4	14·5	4·71	1·4	4·9	6·2	7·4	7·4	9·3

BL, Blood and lymphatic system; BR, Breast; FGO, Female genital organs; GI, Gastrointestinal system; HCV, Heart and central vascular system; MGO, Male genital organs; MS, Musculoskeletal system; NS, Nervous system; OBS, Obstetrics; PV, Peripheral vascular system; RS, Respiratory system; RU, Renal and urinal system; SS, Skin and subcutaneous tissue.

INT, mean interval between hospital admission and onset of the SSI; IQR, interquartile range; LA, mean length of hospitalization of all patients; LN, mean length of hospitalization of patients who acquire an SSI; SSI, surgical site infection; UNK, Unknown.

### Prevalence simulations

**Fig. 2.**
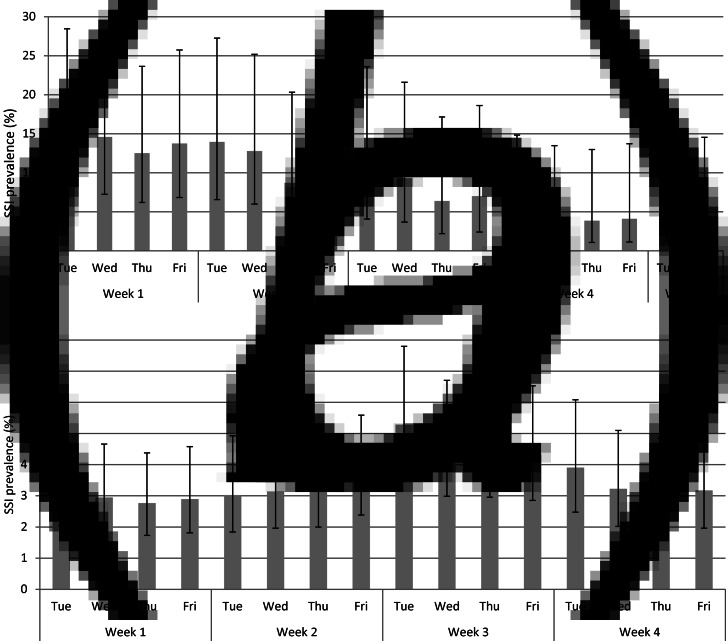
Daily prevalence simulated from incidence dataset II (*a*) at a single hospital in March 2010 and (*b*) at national level in October 2011.

Daily prevalence simulated from incidence surveillance dataset II showed substantial variation during the month, both at hospital and national levels (Fig. 2). As expected, the daily variation in prevalence was larger for individual hospitals. Estimates from other hospitals and national survey months yielded results comparable to the ones presented.

### Rhame & Sudderth method

**Fig. 3.**
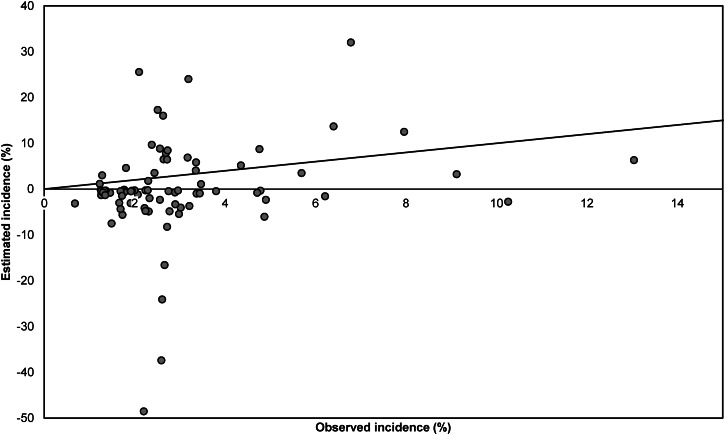
Comparison of observed and estimated incidence of surgical site infections (SSIs) per year at hospital level, for all reported SSIs (dataset I). Estimated incidence was calculated using the Rhame & Sudderth method. One extreme pair of points (observed incidence 7·1%, estimated incidence 105·5%) is not displayed. The diagonal line represents the situation in which the observed and estimated incidence are equal.

**Fig. 4.**
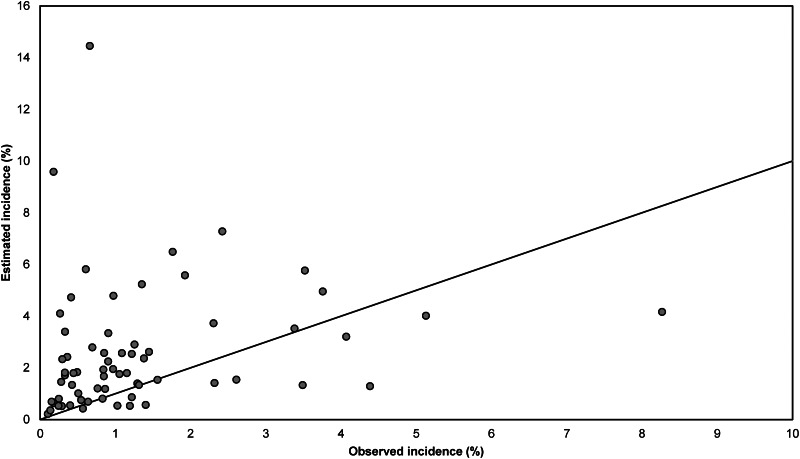
Comparison of observed and estimated incidence of surgical site infections (SSIs) per year at hospital level, for SSIs occurring during the initial hospital stay (dataset II). Estimated incidence was calculated using the Rhame & Sudderth method. Two extreme pairs of points (observed incidence 0·5%, estimated incidence 32·7%; and observed incidence 0·4%, estimated incidence 25·6%) are not displayed. The diagonal line represents the situation in which the observed and estimated incidence are equal.

Estimates of SSI incidence according to the Rhame & Sudderth method were in most cases lower than the incidence surveillance observations based on all reported SSIs (dataset I), and only five (6·1%) out of 82 estimates fell within the 95% confidence interval (CI) for the observed incidence (Fig. 3). The Spearman correlation coefficient between estimated and observed incidence was 0·22, indicating a very weak association. The results presented in Figure 4 are analogous to those of Figure 3 but are based on only those SSIs diagnosed during initial hospital stay (dataset II). Out of 70 estimates, 29 (41%) fell within the 95% CI for the observed incidence. The Spearman correlation coefficient between estimated and observed incidence was 0·35.

### Incidence prediction model

**Fig. 5.**
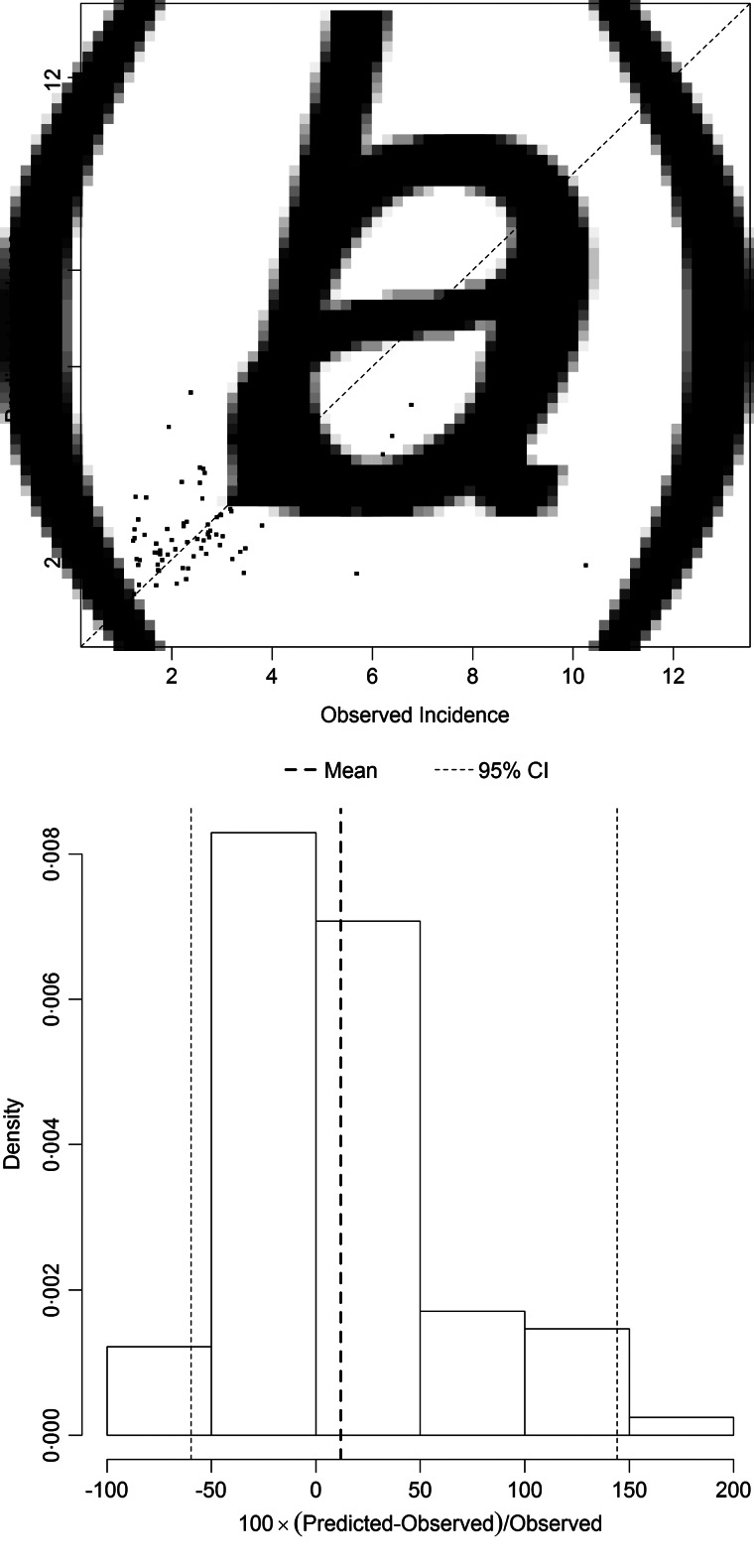
(*a*) Comparison of observed and predicted surgical site infection (SSI) incidence and (*b*) distribution of the percental prediction error, illustrating the performance of the prediction model best predicting SSI incidence (dataset I). The diagonal line in (*a*) represents the situation in which the observed and predicted incidence are equal. The vertical dotted lines in (*b*) display the mean percental prediction error (in bold) and its 95% prediction interval.

**Fig. 6.**
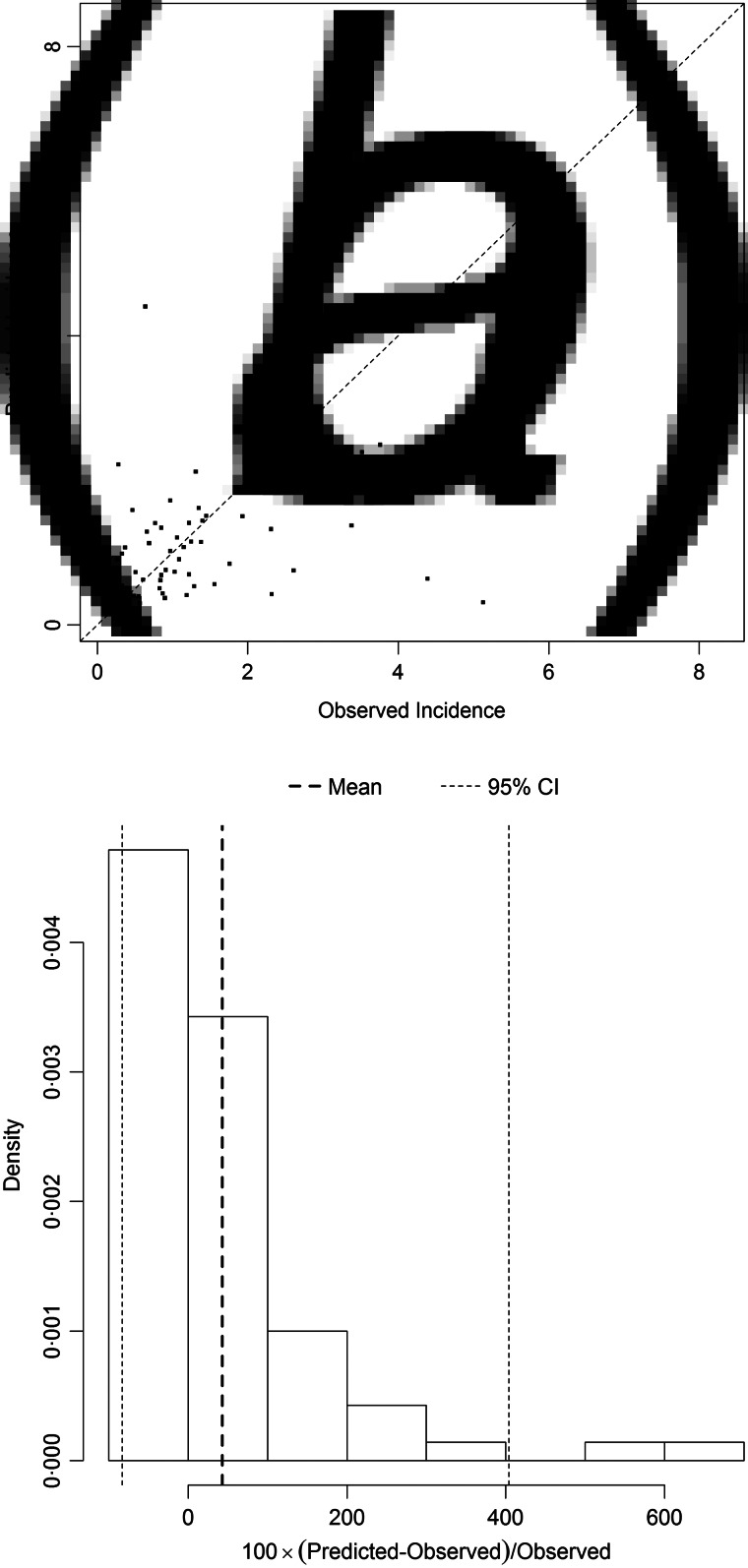
(*a*) Comparison of observed and predicted surgical site infection (SSI) incidence and (*b*) distribution of the percental prediction error, illustrating the performance of the prediction model best predicting incidence of SSIs occurring during the initial hospital stay (dataset II). The diagonal line in (*a*) represents the situation in which the observed and predicted incidence are equal. The vertical dotted lines in (*b*) display the mean percent prediction error (in bold) and its 95% prediction interval.

Figures 5 and 6 show the results of the models best predicting total SSI incidence (dataset I) and incidence of SSIs during initial hospital stay (dataset II), respectively. Variables included in the best performing models were prevalence, LA, LN, gender and hospital as random effect. The performance of the models was quantified on the log scale with the proportion of explained variance, which was 0·27 for total SSI incidence and 0·24 for SSIs during initial hospital stay. The mean percent difference in the model for incidence of all SSIs was 12%, with a 95% prediction interval ranging from –60% to 145%. For the model on SSIs during initial hospital stay only, the mean percent difference was 43%, with a 95% prediction interval ranging from –85% to 405%.

## DISCUSSION

This study showed that HAI prevalence survey data from Dutch hospitals are not suitable to reliably estimate SSI incidence, either by the existing model of Rhame & Sudderth or by our newly developed prediction model including several predictive factors. A first indication of the limitations of prevalence data to estimate SSI incidence was given by the daily fluctuations in the simulated SSI prevalence within a 1-month period. However, as these analyses were based on data including only a subset of specialities, the variability of the true (unselected) SSI prevalence is probably smaller, as variability decreases with higher numbers. Nevertheless, our results at the national level indicate that daily prevalence fluctuates substantially even when the number of patients is large.

When we estimated SSI incidence from prevalence with Rhame & Sudderth's formula, correlation with the observed SSI incidence was poor. Moreover, the incidence estimations based on all SSIs greatly underestimated the incidence surveillance observations and were frequently smaller than zero. This could partly be explained by the fact that from the prevalence surveys we included only the SSIs that occurred during the initial hospital stay, and therefore underestimated the actual prevalence. However, in addition, the incidence estimates smaller than zero were caused by the assumption in the Rhame & Sudderth method that all infections occur in the hospital. The method hereby assumes that patients have surgery and either stay in the hospital until they develop an infection, or go home without complications. The term ‘LN-INT’ in the formula, which describes the period in which patients are admitted to the hospital with an infection, is then a proxy for the duration of infection. However, when the majority of SSIs occur post-discharge and therefore do not influence the initial length of hospitalization, as was the case in our study, this term becomes meaningless. The Rhame & Sudderth method is therefore not applicable to the present situation in Dutch hospitals. When we applied the formula including only SSIs occurring during initial hospitalization, a higher percentage of estimated incidences fell within the range of the incidence surveillance observations, but the correlation between both was still poor. These results confirm our findings that the formula is not adequate for estimating SSI incidence.

In the multilevel prediction models we could not find any combination of (risk) factors that reliably predicted SSI incidence. A possible explanation for the poor performance of the models is that incidence at the hospital level depends heavily on the type and number of reported specialities (as selected by the hospital). For instance, a high-risk surgery (e.g. colectomy) will increase the overall incidence, whereas a low-risk surgery (e.g. total hip replacement) has a reducing effect. Another explanation is that the included risk factors are not associated with SSI to the same extent in all specialities, for which reason the impact of a factor on a speciality might be eliminated by the lack of impact on another. Taking these remarks into account, it might not be possible to predict SSI incidence at the hospital level. However, due to the small numbers of patients and infections per speciality in the prevalence surveys, it was not possible to extend the model to speciality- or procedure-specific SSI incidence predictions.

A considerable number of studies have used the Rhame & Sudderth method to calculate HAI incidence from prevalence survey data [13, 20, 21, 23, 32–34], but only few have rigorously investigated the applicability of the method for SSIs [21, 24]. In a report on the 2007 Scottish national HAI prevalence survey, SSI incidence was estimated per surgical category [24]. Because of small numbers, only three categories had sufficient data for comparison. The results were highly variable and included no reliable estimates; however, Gastmeier *et al.* did find promising results [21]. For eight hospitals combined, estimated SSI incidence and prevalence were within the 95% CI of the corresponding observed rate. However, their study differed from ours in several aspects. First, their study was performed as part of an infection control management study for all HAIs, including three prevalence surveys and incidence surveillance on all patients discharged during an 8-week period. Second, only infections that developed during hospital stay were reported, which does not adequately reflect actual surveillance results. In addition, Gastmeier *et al*. showed the incidence estimates for individual HAI, including SSIs, only for all hospitals combined. These results indicate that combining outcomes of repeated prevalence surveys may reduce variability. A study by Ustun *et al*. using the mean prevalence from weekly surveys also demonstrated a close relationship between prevalence and incidence [13]. If hospitals had performed several (or at least more than two) prevalence surveys per year, we might have found similar results. Our study, however, focused on estimating SSI incidence from prevalence surveys in the current ongoing nationwide programme, reflecting daily practice, rather than from multiple surveys that were part of a single short-term study. To the best of our knowledge there are no published alternative methods to calculate or predict SSI incidence for data aggregated at hospital level. Other prediction models for SSIs exist, but these are aimed at predicting the risk for individual patients [35–38].

This study has several important strengths. Data were derived from a national surveillance network that uses a standardized protocol with strict definitions to diagnose SSIs and mandatory onsite validation every 3–5 years, resulting in high-quality surveillance data. For this study we only included hospitals participating in both incidence surveillance and prevalence surveys simultaneously, which resulted in the most optimal link between the two.

There were also some limitations to this study. Although strict protocols were implemented, routine surveillance data is typically not collected for study purposes, resulting in some important factors that were not collected in the best way. In the incidence surveillance, the type of surgery was collected, whereas only the patient's speciality was reported in the prevalence surveys. We have taken this into account as much as possible by linking hospital data at the speciality level, in order to make for the greatest comparability between incidence and prevalence. Furthermore, because dates of discharge were not available from prevalence surveys, the estimates of LA, LN and INT were derived from incidence surveillance. It is difficult to assess the impact this had on the results of Rhame & Sudderth's formula, especially for the analysis of total SSI incidence where the SSIs that develop post-discharge led to meaningless values for the duration of infection (‘LN-INT’). However, when only SSIs during the initial hospital stay were included, LA, LN and INT most likely were lower than when prevalence data would have been used, since prevalence surveys are biased towards patients with longer lengths of stay. How this impacted on the outcomes is highly dependent on the ratios between the values. The used values of LA, LN and INT might have influenced the performance of the prediction models as well, but their impact was likely to be limited. Finally, hospitals are free to choose which types of surgery they want to include in their incidence surveillance and may change their selection annually. This means that the distribution of surgery types in the incidence surveillance is not a reflection of the true distribution in the hospital, and therefore the calculated SSI incidence at hospital level is only based on a selection of the total number of specialities in the hospital.

In conclusion, SSI incidence cannot be reliably predicted from SSI prevalence survey data using the currently available methods. This is caused by (i) the design of the prevalence surveys, which give only a snapshot of the current infection status of admitted patients, (ii) the large number of SSIs that occur after the initial hospitalization, and (iii) the infection duration of SSIs which is difficult to estimate and cannot be reliably approximated by the ‘time from infection until discharge’. We therefore conclude that prevalence surveys, as currently implemented in The Netherlands, are not a useful measure to estimate SSI incidence and that they cannot replace SSI incidence surveillance in order to reduce the workload and expenses for HAI surveillance. Although these findings are likely to be universal, further research should be performed to investigate whether this also applies for other countries with similar prevalence survey protocols [14, 15]. For infection prevention purposes, both types of surveillance will remain important and are in fact complementary. Prevalence surveys can give a first indication of the areas of interest for infection control and are useful in visualizing trends, while incidence surveillance is better equipped when more in-depth information is needed. When choosing a surveillance method, hospitals should always be aware of the value of both surveillance systems and keep in mind the goals they aim to achieve.
